# Crystal digital droplet PCR for detection and quantification of circulating *EGFR* sensitizing and resistance mutations in advanced non-small cell lung cancer

**DOI:** 10.1371/journal.pone.0183319

**Published:** 2017-08-22

**Authors:** Cécile Jovelet, Jordan Madic, Jordi Remon, Aurélie Honoré, Romain Girard, Etienne Rouleau, Barbara André, Benjamin Besse, Magali Droniou, Ludovic Lacroix

**Affiliations:** 1 Plateforme de Génomique-BMO et Centre de Ressources Biologiques, AMMICA, INSERM US23/CNRS UMS3655, Gustave Roussy, Villejuif, France; 2 Stilla Technologies, 1 Mail du Professeur Georges Mathé, Villejuif, France; 3 Département de Médecine Oncologique, Institut Gustave Roussy, Villejuif, Faculté de médecine, Université Paris Sud, Le Kremlin-Bicêtre, France; 4 Ecole Polytechnique, Route de Saclay, Palaiseau, France; 5 Département de Biologie et Pathologie Médicales, Institut Gustave Roussy, Villejuif, France; Universitat de Barcelona, SPAIN

## Abstract

Over the past years, targeted therapies using tyrosine kinase inhibitors (TKI) have led to an increase in progression-free survival and response rate for a subgroup of non-small cell lung cancer (NSCLC) patients harbouring specific gene abnormalities compared with chemotherapy. However long-lasting tumor regression is rarely achieved, due to the development of resistant tumoral subclones, which requires alternative therapeutic approaches. Molecular profile at progressive disease is a challenge for making adaptive treatment decisions. The aim of this study was to monitor *EGFR*-mutant tumors over time based on the quantity of mutant DNA circulating in plasma (ctDNA), comparing two different methods, Crystal™ Digital™ PCR and Massive Parallel Sequencing (MPS). In plasma circulating cell free DNA (cfDNA) of 61 advanced NSCLC patients we found an overall correlation of 78% between mutated allelic fraction measured by Crystal Digital PCR and MPS. 7 additional samples with sensitizing mutations and 4 additional samples with the resistance mutation were detected with Crystal Digital PCR, but not with MPS. Monitoring levels of both mutation types over time showed a correlation between levels and trends of mutated ctDNA detected and clinical assessment of disease for the 6 patients tested. In conclusion, Crystal Digital PCR exhibited good performance for monitoring mutational status in plasma cfDNA, and also appeared as better suited to the detection of known mutations than MPS in terms of features such as time to results.

## Introduction

Lung cancer is the third most frequent cancer and the leading cause of cancer related death worldwide and non-small cell lung cancer (NSCLC) accounts for approximately 80% cases of lung cancer [1]. About 16% of non-small-cell lung cancers (NSCLC) are diagnosed at localized stage, where surgery remains the standard treatment, while 22% are diagnosed at locally advanced disease and up to 60% in advanced stage [2]. Conventional chemotherapy is frequently confronted to resistance mechanisms in advanced NSCLC due to the selection of molecular alterations in tumor cells. The introduction of targeted therapies has changed the treatment paradigm and has established tumor genotyping as an essential routine diagnostic tool in clinical practice. In Caucasian population, *EGFR* activating mutations account for approximately 11% of genetic alterations in advanced NSCLC, mainly adenocarcinoma [3]. In patients harbouring sensitizing *EGFR* mutations, especially small in-frame deletion/insertions in exon 19 (*EGFR* Del19) and p.L858R mutation in exon 21, inhibition of *EGFR* kinase activity by tyrosine kinase inhibitors (EGFR-TKIs) improves response rate and progression-free survival compared to standard first-line platinum doublet chemotherapy, making them the standard of care [4,5]. *EGFR* mutations profiling is therefore a critical step in identifying the patients with increased sensitivity to EGFR-TKIs treatment [6]. Unfortunately, almost all patients will develop acquired resistance to EGFR-TKIs. Mechanisms of acquired resistance may be categorized as: (1) secondary *EGFR* mutations, such as mutations in exon 20 (2) bypass track signalling pathways like *MET* or *HER2* amplification, or (3) histologic transformation mainly to small-cell carcinoma with or without *PI3KCA* mutation [7]. The substitution of threonine to methionine at codon 790 (p.T790M) in exon 20 of the *EGFR* gene decreases sensitivity to first-generation EGFR-TKIs and accounts for over half of resistance mechanisms [7–9]. Osimertinib (AZD9291), a third-generation EGFR-TKI with activity against sensitive and resistant *EGFR* mutations, is the standard treatment in *T790M*-positive NSCLC patients after disease-progression on EGFR-TKIs [10], and it has received the approval by FDA and EMA. Recent studies [10,11], have reported the feasibility of liquid biopsies for testing T790M and personalising treatment among lung cancer patients; and efficacy of osimertinib is equivalent irrespective if T790M-positive status is detected in tissue or liquid biopsy [10–12].

Development of analysis performed on plasma circulating cell free DNA (cfDNA) offers a blood-based minimally-invasive alternative to tissue biopsy [13–14]. Indeed, potential applications of cfDNA based analysis, even when tissue sample is not available, include molecular abnormalities characterization opportunity at diagnosis, patient selection for targeted therapies, prognostic assessment, monitoring of treatment response and resistance, as well as screening for acquired mutations at the time of resistance [15]. Currently, detection of mutated circulating DNA remains a challenging analytic owing to its occurrence in small fragments and its low proportion of tumor mutated cfDNA (ctDNA) in total circulating cell-free DNA (cfDNA) [13]. However, it is not well established what is the best technique for molecular profiling in liquid biopsies. Recent improvement of PCR-based methods, massive parallel sequencing (MPS) and digital PCR now enable reliable detection and quantification of mutation in cfDNA, thereby opening up possibilities for clinical use. Digital droplet PCR (dPCR) involve partition of target DNA templates into water in oil emulsion followed by PCR amplification and end-point quantification of amplified partitions using fluorescent signal, showing high specificity and sensitivity for detecting and quantifying cancer mutations [16–18]. Recent French recommendations, by GFCO based on expert discussion, proposed determination of *EGFR* mutational status based on cfDNA in second intention at diagnostic, when tissue is not available, and in first intention for patient with progression under EGFR-TKIs therapy [19].

In the current study, we assessed a newly developed digital PCR platform, the Naica system for Crystal Digital PCR, to detect and quantify *EGFR* sensitizing and resistance mutations in the blood of advanced *NSCLC* patients. This platform generates 2D arrays of monodispersed droplets in a microfluidic chip which is subsequently thermocycled, then imaged using a fluorescent microscope. Taking advantage of the 3 distinct fluorescence channels available, we designed multiplex assays for the concomitant detection and quantification of wild-type and *EGFR* Del19, p.L858R, p.L861Q, or p.T790M mutations. We investigated this method in a prospective cohort of advanced *NSCLC* patients including longitudinal samples and we performed a comparison with results obtained on the same samples using massive parallel sequencing.

## Patients and methods

### Patients and sample collection

We prospectively investigated the *EGFR* mutational status in cfDNA of 61 advanced NSCLC patients and for whom re-biopsy was not feasible. A total of 50 patients had proven targetable *EGFR* mutations detected in non-synchronous tumor tissue by MPS and 11 additional patients without any tumor *EGFR* mutation were included to assess the specificity of the methods. The *EGFR* mutational status was tested either in EGFR-TKI naive patients or in patients on EGFR-TKI treatment. Digital PCR and MPS analysis were conducted in blood samples from eligible patients treated at the Gustave Roussy cancer center (Villejuif, France) from June 2015 to April 2016. All patients provided written informed consent for biomedical research (CEC-CTC IDRcb2008-AOO585-50) and the institutional ethics committee approved the protocol. Among the 61 patients tested, 14 patients had at least one follow-up sample in order to detect new mutations or to monitor mutations detected at baseline and 7 patients had at least 3 samples.

### Extraction and quantification of cfDNA

Blood samples (10 ml) were collected in EDTA-K2 tubes (BD Vacutainer–Beckton), Dickinson and Company, Franklin Lakes, NJ) and centrifuged for 10 minutes at 1000 g within maximum 4 hours after the blood withdraw. Then plasma was further centrifuged at 14,000 g for 10 minutes at room temperature and stored at −80°C until analysis. DNA was extracted from 3 mL of plasma using the QIAamp circulating nucleic acid kit (Qiagen) according to the manufacturer’s instructions, and resuspended in 40 μL of AVE buffer. A real-time quantitative PCR TaqMan™ assay targeting *GAPDH* was used to measure plasma DNA concentration.

### Detection of *EGFR* mutations by Crystal^TM^ Digital PCR

**Fig 1:**
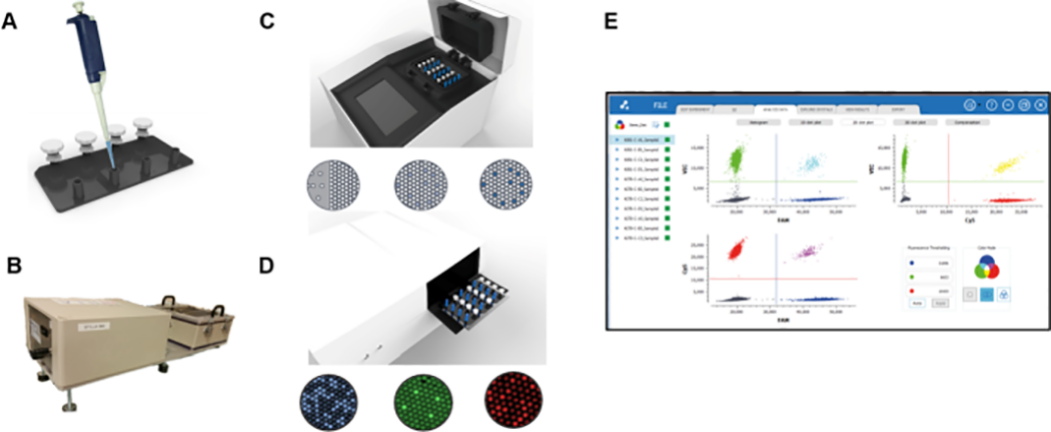
The Naica System for Crystal Digital PCR. The system comprises Sapphire Chips v1 (A.), in which the sample is partitioned as droplet crystals using the Stilla loading device (B), the Naica Geode (C), which will perform thermal cycling, the Naica Prism3 (D), which enables the fluorescent readout of the droplet crystals in three detection channels (Blue, Green and Red), and finally, the Crystal Miner analysis software (E) for data extraction and concentration calculations from images acquired with the Prism3.

Detection of *EGFR* mutation of interest in ctDNA was done on the Naica digital PCR system (Stilla Technologies, France) (**Fig 1**). Development of the quadriplex dPCR assay for the detection of mutation p.L858R, p.L861Q and p.T790M in *EGFR gene (reference sequence NM_005228*.*3)* was described previously [20]. Primers and probes for the detection of small in-frame deletion/insertions in exon 19 have been described elsewhere [17,18] and combined with p.T790M PCR model in a second multiplex dPCR assay. Digital PCR reactions were assembled using PerFecTa Multiplex qPCR ToughMix (Quanta Biosciences, Gaithersburg, MD, USA), 40nM FITC (Saint Louis, MO, USA), 1μl of primer and probes multiplex mix and 3 μl of DNA template. Sapphire prototype (v.1) chips (Stilla Technologies, Villejuif, France) were first primed with PCR oil using the Stilla-loading device. A total of 4 PCR reactions of 20μl each were then loaded per Sapphire chip before being compartmentalized into 15,000 to 20,000 droplets using the Stilla loading device. Finally, the inlet and outlet ports of the Stilla chips were overlayed with Capping oil (Stilla Technologies), prior to thermocycling using the Naica Geode prototype thermocycler. Cycling conditions were 95°C for 10 minutes, followed by 45 cycles of 95°C for 10 seconds and 62°C for 15 seconds. Sapphire chips containing the 2D crystals of droplets generated were imaged using the Naica Prism3 reader and fluorescent data were analyzed using Crystal Miner software (Stilla Technologies). Each patient sample was tested in duplicate. Standard wild-type DNA and standard mutated DNA (Horizon Discovery, Cambridge, UK) were used as negative and positive controls respectively. Negatives and positives droplets were discriminated using manual thresholding according to the signal given by the negative and positive controls included in each individual experiment.

### Detection of *EGFR* mutations by MPS

Massive parallel sequencing analyses were conducted as previously [14]. Targeted sequencing libraries were generated using the Ion AmpliSeq Library kit 2.0 according to the manufacturer’s instructions (Life Technologies, Darmstadt, Germany). Plasma samples were analyzed independently with Cancer Hotspot Panel v2 (CHP2) targeting 50 cancer genes covered by 207 amplicons (Life Technologies). The primers used for amplification were partially digested by FuPa enzyme. The digested product was then ligated with adapters and barcodes, then amplified and purified using AMPure XP PCR purification (Beckman Coulter) according to manufacturer recommendations. The purified libraries were quantified using the Qubit 2.0 Fluorometer (Invitrogen). Equal amounts of each library were pooled, emulsified and PCR amplified with the Ion OneTouch 2 system using the Ion PGM^TM^ Template OT2 200 Kit (Life Technologies). The enrichment was then performed with the Ion One Touch ES (Enrichment System) and the enriched Ion Spheres were loaded into a 316v.2 Ion Sequencing Chip. Sequencing data were analyzed using the Torrent Suite Variant Caller 4.2 software and reported somatic variants were compared to the reference genome hg19. The variants were called if >5 reads supported the variant and/or total base depth >50 and/or variant allele frequency >1% was observed. All the variants identified were visually controlled on.bam files using Alamut Visual v2.8.x software (Interactive Biosoftware, Rouen, France). All the germline variants found in 1000 Genomes Project or ESP (Exome Sequencing Project database) with frequency >0.1% were removed. All somatic mutations were annotated, sorted and interpreted by an expert molecular biologist according to available databases (COSMIC, TCGA). In the current study, only *EGFR* mutations were reported in results section.

## Results

### Patients’ characteristics

Patients characteristics at the time of baseline blood sampling (Table 1) were as follow: the median age was 62 years (range: 37–83 years). 43 (70%) were female. 32 patients were never smokers (52%), 6 were current smokers (10%) and 23 were former smokers (38%). The median number of previous treatment lines was 2 (range: 0–11).

**Table 1:** Patient characteristics.

	Total *N* = 61
Age	
median (range)	62 (37–83)
Gender	
Male	18
Female	43
Smoker status	
never smoker	32
current smoker	6
former smoker	23
Number of previous lines of therapy	
median (range)	2 [0–11]

### Determination of the limit of blank of multiplex dPCR assays

The limit of blank (LOB) was measured for each target detected in the 2 multiplex assays. A total of 32 independent dPCR assays were performed on human wild-type DNA ranging from 639 to 12393 copies per dPCR reaction, representing 2.1 to 40.9 ng of DNA. No correlation was found between the quantity of wild-type DNA in dPCR reaction and the number of false positives (no statistical significance under Spearman's rho test; data not shown). The mean number (μ) of non-expected positives droplets occurring in the wild-type controls was measured and found to fit a Poisson distribution. The LOB with 99% confidence level was defined as the smallest number of events (positive droplets) at which the cumulative Poisson distribution with parameter μ exceeding 99%. Thus, the LOB at 99% was equal to 2 positive droplets for all EGFR mutations except for the second multiplex assays of the T790M mutation (S1 Table). For all assays, the sample was considered as positive if the number of positive droplets was strictly larger than the LOB at 99%.

### Detection of *EGFR* mutations using dPCR and MPS in plasma samples

**Fig 2:**
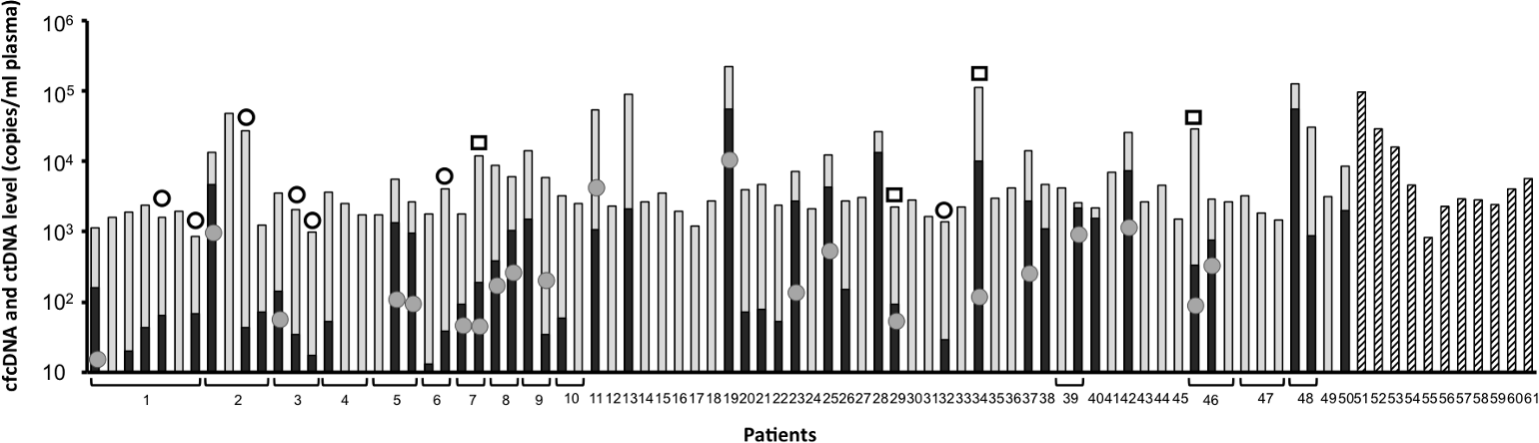
Wild-type and mutant DNA levels measured by dPCR in 87 plasma samples from 61 metastatic NSCLC patients. Greyed bar: circulating cell-free DNA (cfDNA) concentration in NSCLC patients with confirmed targetable EGFR mutations in tumor tissue. Filled bar: sensitizing (Del19, p.L858R and p.L861Q) mutations concentration. Dashed bar: wild-type DNA concentration in NSCLC patients with wild-type EGFR in tumor tissue. Grey dots: p.T790M mutation concentration. Empty circles and squares indicates sensitizing and resistance mutations positives by dPCR but not detected by NGS respectively.

A total of 87 plasma samples derived from 61 advanced NSCLC patients were analyzed in this study (Fig 2, S2 Table). Initial *EGFR* mutational status reported in this work were characterized in non-synchronously collected diagnostic tumor tissue samples. An *EGFR* activating mutation (Del19, p.L858R or p.L861Q) was found in tumor DNA of 50 patients (38 Del19, 10 p.L858R and 2 p.L861Q). Among this 50 patients, 31 (62%) had the initial activating mutation detectable in plasma. Concomitant *EGFR* p.T790M mutation was found in 17 (55%) patients.

**Fig 3:**
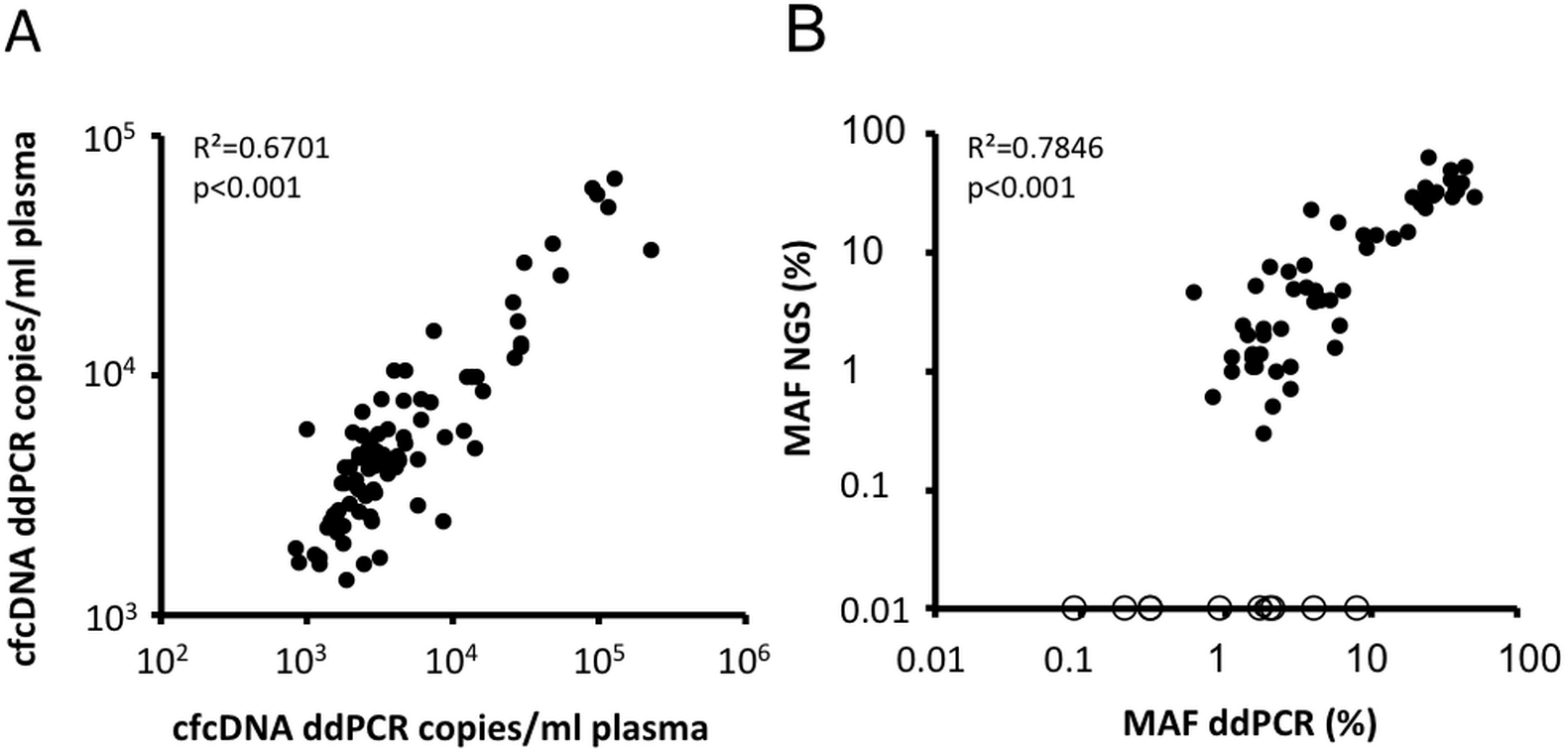
A. Correlation between total circulating cell-free DNA copies per ml of plasma measured by dPCR and qPCR in 87 plasma samples of 61 metastatic NSCLC patients. B. Correlation between mutant allele fraction (MAF) measured for sensitizing and resistance mutations by dPCR and NGS. Empty circles represent samples not detected by NGS.

Circulating cell-free DNA (cfDNA) concentration measured by quantitative PCR and dPCR were correlated and ranged from 827 to 225735 copies/ml plasma (median 2972 copies/ml of plasma) (Fig 3A, S2 Table). Mutated cfDNA (ctDNA) concentration measured by dPCR ranged from 12.6 to 58821 copies/ml of plasma and mutant allele fraction (MAF) was found to range from 0.09 to 51.1% of total cfDNA. Measures of mutant allele fraction using dPCR and MPS displayed significant correlation (Fig 3B).

Overall, *EGFR* sensitizing mutations were detected in 46 plasma samples following dPCR analysis whereas MPS detected 39 *EGFR* sensitizing mutations in the same plasma samples. The p.T790M mutation was detected in 21 plasma samples (17 patients) using dPCR and 17 plasma samples (15 patients) using MPS. The detection of p.T790M mutation in plasma has resulted in the change of treatment into osimertinib for some patients. No *EGFR* mutation was detected by any of the two methods in the 11 plasma samples derived from *EGFR* WT patient. No plasma sample was positive following MPS analysis and negative using dPCR. The 11 samples positives by dPCR but not detected by MPS had low mutant allele fraction ranging from 0.09% to 7.9% (Fig 3B, S2 Table).

### Monitoring of *EGFR* mutations in longitudinal samples

**Fig 4:**
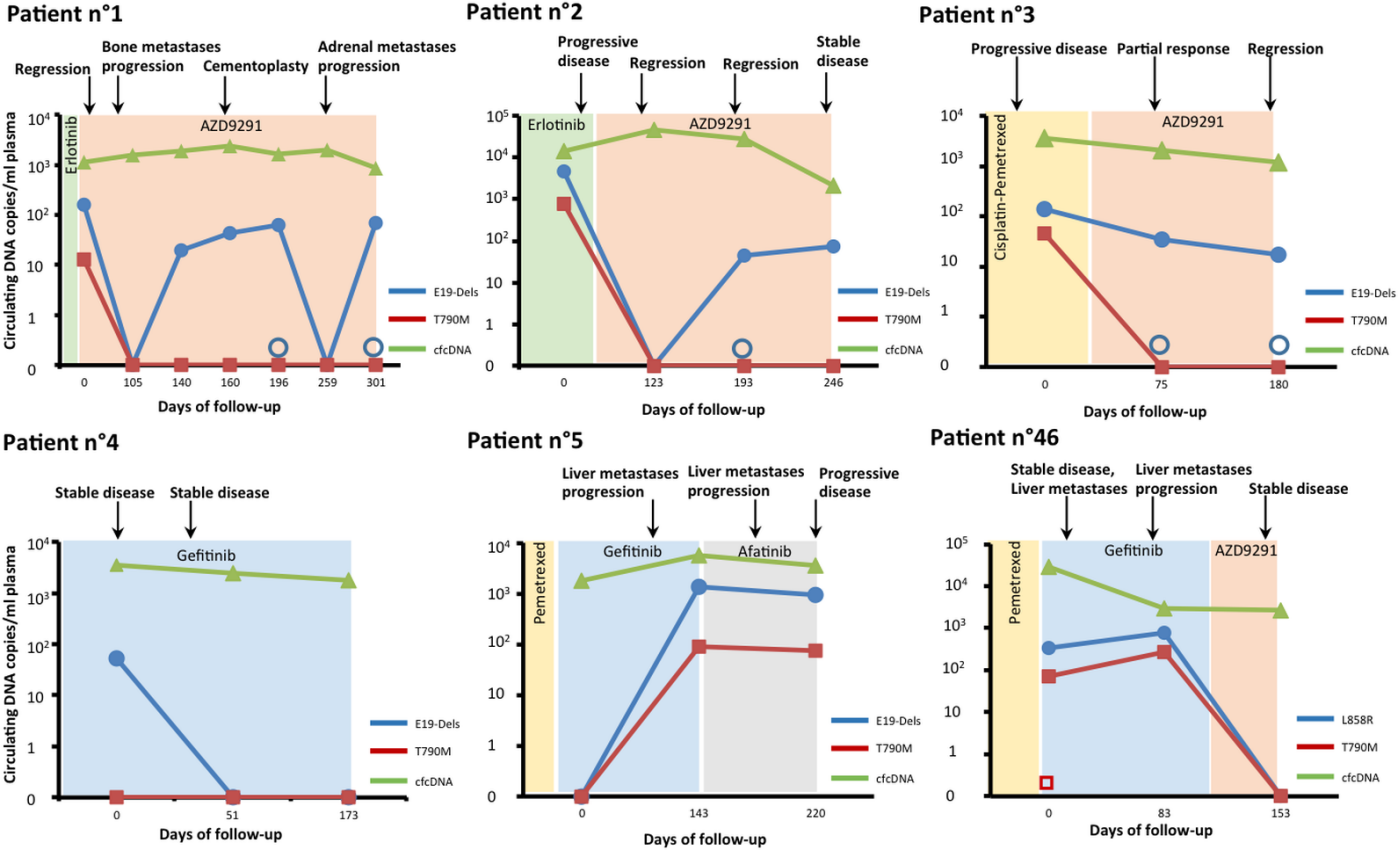
Monitoring of targeted sensitizing and resistance EGFR mutations and circulating cell-free DNA levels over time in 6 metastatic NSCLC patients using dPCR. Coloured region indicates period of chemotherapy. Radiological assessment of patient response is indicated above the figures. Empty circles and squares indicates sensitizing and resistance mutations positives by dPCR but not detected by NGS respectively.

Seven patients had a minimum of 3 blood samples collected during follow-up. ctDNA was detected in 6 out of the 7 patients and was reported as well as clinical observations, radiological assessments, line of chemotherapy and concentration of cfDNA (Fig 4). The level of ctDNA reflected well the course of the disease. In contrast, cfDNA concentration did not vary according to the course of the disease. Levels of sensitizing mutation remained below 10^2^ copies/ml plasma and/or decreased for patients 1, 2, 3, 4 and 46 who displayed tumor regression or with stable disease. In patients 1, 2, 3 and 46 treated with osimertinib, decrease of T790M mutation level was observed. In contrast, in patient 5 treated with first generation EGFR-TKIs, disease progression was observed concomitantly with increasing and/or higher levels of sensitizing and resistance mutations. Six plasma samples were negatives using MPS analysis whereas dPCR yielded positive results highlighting the need for sensitive technics in order to avoid the loss of precious data for clinical management of patients.

## Discussion

Crystal Digital PCR exhibited good performance for monitoring *EGFR* mutational status in plasma cfDNA, and also appeared as better suited to the detection of known mutations than MPS. This is important in advanced NSCLC patients for whom selection for targeted therapies using EGFR-TKI is based on the presence of *EGFR* actionable mutations and therefore requires tumor genotyping. Acquired secondary resistance to EGFR-TKI treatment following T790M mutation is the main mechanisms of acquired resistance. When biopsy is not available or not feasible, detection and characterization of *EGFR* mutation could be achieved using cfDNA based analysis according to current recommendations for management of NSCLC patients [19,21]. Moreover, approaches based on cfDNA analysis provide additional information, as cfDNA concentration or *EGFR* mutation MAF, that would be useful for the monitoring of disease or treatment response and could avoid continuing ineffective therapies. Despite challenging, the quantification of ctDNA was greatly improved by methods such as BEAMing, ultra-sensitive massive parallel sequencing and digital PCR (dPCR) [22–24]. Here, we used a newly released dPCR platform, the Naica system for Crystal Digital PCR to monitor levels of *EGFR* mutations in cfDNA of NSCLC patients. We also presented a clinically relevant use of this method through monitoring of cfDNA in longitudinal samples during follow-up. To our knowledge, this is the first droplet digital PCR platform which enables multiplex detection of targets in 3 independent fluorescent channels. This manuscript reports the major advantages of Crystal Digital PCR, which are its enhancing sensitivity and higher multiplex capacity.

By combining dedicated fluorescent probes and primers, we optimized 2 multiplex assays for the detection and quantification of small in-frame deletion/insertions in exon 19, p.T790M and WT *EGFR* DNA copies) and 4 (p.L858R, p.L861Q, p.T790M and WT *EGFR* DNA copies) *EGFR* targets respectively. The p.L858R and p.L861Q mutations were detected using the same fluorescent channel. To estimate the limit of blank (LOB) with 99% confidence level of the assays, replicates containing various quantity of human wild-type only were performed. We did not observe any correlation between wild-type DNA fragments level and occurrence of false positives droplets, in agreements with observations reported by Zonta *et al*. [25] when using TaqMan probes. A sample was considered positive when the number of positive droplets obtained was strictly superior to the LOB measured with the corresponding test. The multiplex dPCR assays enable detection of *EGFR* mutations in samples with mutant allele fraction (MAF) as low as 0.09% whereas massive parallel sequencing enable detection of MAF down to 0.3%. In addition, several samples with low MAF were not detected using MPS analysis.

Regarding performance of *EGFR* mutational status detection in plasma samples collected non synchronously to diagnostic, we detect initial *EGFR* mutation in 38 of 50 patients (62%) with mutation known in tissues. No discordance in type of mutation was observed for the 61 cases. This observation is in accordance with already published data resumed in Qiu *et al*. meta-analysis of 22 studies [26], with a pooled sensitivity of 62% and a pooled specificity of 95.6%.

In follow-up samples, the better sensitivity of dPCR enabled a more accurate monitoring of *EGFR* mutations and reflected well the evolution of the disease along the treatments. Those results demonstrate the strength of this assay to track *EGFR* mutation during the course of EGFR-TKI treatment. The cfDNA based tests are optimal to monitor biomarkers of sensitivity or resistance associated with targeted therapies in lung cancer; mainly when tissue in not available and biopsy is not indicated. Osimertinib has been approved for patients with metastatic NSCLC tested positive for the *EGFR* p.T790M mutation, occurring during disease progression or after EGFR-TKI therapy, when additional tissue biopsy is not always achievable without risk. The proposed assay can be used for the follow-up of NSCLC patients, and the optimization of EGFR-TKIs treatment.

The Crystal Digital PCR method appeared as better suited to the detection of known mutations than MPS in terms of features such as cost and time to results. Nevertheless, the reported assay is limited to most prevalent *EGFR* mutations detection and is not aiming to detect other mechanisms of acquired resistance on EGFR-TKI such as MET or HER2 amplifications [7]. But additional probes for MET/ HER2 amplification detection could be design and will deserve additional study with samples from patient developing this kind of resistance mechanism to determine added value of such additional assay.

In conclusion, this study demonstrated that Crystal Digital PCR is a fast and sensitive technology enabling ctDNA detection and quantification, specifically for *EGFR* mutations. Early and frequent cfDNA analysis should provide molecular information for lung cancer therapeutic management, improving number of patients that can get benefit of personalized treatment, especially when tumor tissue is not available.

## Supporting information

S1 Table Determination of the limit of blank (LOB) of the multiplex PCR assays. Detection assays were performed on standard WT DNA. The number of non-expected positives droplets was fitted to a Poisson distribution (p-values show that Poisson hypothesis cannot be rejected under the chi-square goodness of fit test). The LOB in number of droplets was derived from one-tailed 99% upper limit of the cumulative Poisson distribution. Mutation detection models were combined in two distinct multiplex assays marked * and ** respectively.
(DOCX)

S2 Table Quantification of cfDNA and ctDNA levels. Quantification of cfDNA and ctDNA levels in 87 plasma samples of 61 metastatic NSCLC patients. ND: not detected, NA: not available.
(DOCX)
