# Supplementary material for: Crystal digital droplet PCR for detection and quantification of circulating EGFR sensitizing and resistance mutations in advanced non-small cell lung cancer
Source: PLoS One. 2017 Aug 22;12(8):e0183319. doi: 10.1371/journal.pone.0183319 (PMC5567481; doi:10.1371/journal.pone.0183319)
Supplement: S1 Table — Detection assays were performed on standard WT DNA. The number of non-expected positives droplets was fitted to a Poisson distribution (p-values show that Poisson hypothesis cannot be rejected under the chi-square goodness of fit test). The LOB in number of droplets was derived from one-tailed 99% upper limit of the cumulative Poisson distribution. Mutation detection models were combined in two distinct multiplex assays marked * and ** respectively. (DOCX) [file pone.0183319.s001.docx]

**Supplementary Table 1 : Determination of the limit of blank (LOB) of the multiplex PCR assays.** Detection assays were performed on standard WT DNA. The number of non-expected positives droplets was fitted to a Poisson distribution (p-values show that Poisson hypothesis cannot be rejected under the chi-square goodness of fit test). The LOB in number of droplets was derived from one-tailed 99% upper limit of the cumulative Poisson distribution. Mutation detection models were combined in two distinct multiplex assays marked * and ** respectively.

*EGFR* n° specificity n° tests with 0 n° tests with 1 n° tests with 2 Mean (µ) p-value Poisson

mutations test non-expected non-expected non-expected LOB 99%

positive droplet positive droplet positive droplets (n° droplets)

T790M* 32 28 3 1 0.156 0.783 2

L858R/L861Q* 32 21 9 2 0.406 0.989 2

E19-Dels** 32 21 8 3 0.344 0.913 2

T790M** 32 30 1 1 0.094 0.132 1
