# Supplementary material for: Crystal digital droplet PCR for detection and quantification of circulating EGFR sensitizing and resistance mutations in advanced non-small cell lung cancer
Source: PLoS One. 2017 Aug 22;12(8):e0183319. doi: 10.1371/journal.pone.0183319 (PMC5567481; doi:10.1371/journal.pone.0183319)
Supplement: S2 Table — Quantification of cfDNA and ctDNA levels in 87 plasma samples of 61 metastatic NSCLC patients. ND: not detected, NA: not available. (DOCX) [file pone.0183319.s002.docx]

**Supplementary table 2: Quantification of cfDNA and ctDNA levels in 87 plasma samples of 61 metastatic NSCLC patients.** ND: not detected, NA: not available.

|  |  |  |  |  |  | **Mutant EGFR copies/ml plasma by ddPCR** | | | **Mutant allele fraction ddPCR (%)** | | | **Mutant allele fraction MPS (%)** | | |
| --- | --- | --- | --- | --- | --- | --- | --- | --- | --- | --- | --- | --- | --- | --- |
| **Case no.** | **Tumor DNA EGFR mutational status** | **Sampling time (day)** | **Plasma volume analyzed (ml)** | **cfDNA cp/mL plasma by qPCR** | **cfDNA cp/mL plasma by ddPCR** | **L858R/L861Q** | **E19-Dels** | **T790M** | **L858R/L861Q** | **E19-Dels** | **T790M** | **L858R/L861Q** | **E19-Dels** | **T790M** |
| 1 | E19-Dels | 0 | 3.5 | 1794 | 1131 | 0 | 160 | 12.6 | 0 | 14.1 | 1.1 | 0 | 13 | 1 |
|  |  | 105 | 2.3 | 2226 | 1594 | 0 | 0 | 0 | 0 | 0 | 0 | 0 | 0 | 0 |
|  |  | 140 | 3 | 1400 | 1867 | 0 | 20 | 0 | 0 | 1.1 | 0 | 0 | 1.3 | 0 |
|  |  | 160 | 3 | 5627 | 2377 | 0 | 44 | ND | 0 | 1.9 | ND | 0 | 2 | 0 |
|  |  | 196 | 3 | 2667 | 1599 | NA | 64 | 0 | NA | 4 | 0 | 0 | 0 | 0 |
|  |  | 259 | 3 | 2933 | 1927 | NA | 0 | 0 | NA | 0 | 0 | 0 | 0 | 0 |
|  |  | 301 | 3 | 1667 | 865 | NA | 68 | 0 | NA | 7.9 | 0 | 0 | 0 | 0 |
| 2 | E19-Dels | 0 | 3.6 | 9878 | 14168 | 0 | 4700 | 790 | 0 | 33.2 | 5.6 | 0 | 49 | 18 |
|  |  | 123 | 3 | 35267 | 47560 | NA | ND | 0 | NA | ND | 0 | 0 | 0 | 0 |
|  |  | 193 | 3 | 16747 | 27670 | NA | 44 | 0 | NA | 0.2 | 0 | 0 | 0 | 0 |
|  |  | 246 | 3.6 | 1733 | 1226 | NA | 73.3 | 0 | NA | 6.0 | 0 | 0 | 2.4 | 0 |
| 3 | E19-Dels | 0 | 3.7 | 5978 | 3561 | 0 | 142.7 | 45.4 | 0 | 4.0 | 1.3 | 0 | 3.9 | 2.4 |
|  |  | 75 | 3 | 5827 | 2048 | NA | 34.7 | 0 | NA | 1.7 | 0 | 0 | 0 | 0 |
|  |  | 180 | 3.7 | 5978 | 988 | NA | 17.3 | 0 | NA | 1.8 | 0 | 0 | 0 | 0 |
| 4 | E19-Dels | 0 | 3 | 3867 | 3593 | NA | 53.3 | ND | NA | 1.5 | ND | 0 | 1.1 | 0 |
|  |  | 51 | 3 | 3160 | 2480 | NA | 0 | 0 | NA | 0 | 0 | 0 | 0 | 0 |
|  |  | 173 | 3 | 3533 | 1736 | NA | 0 | 0 | NA | 0 | 0 | NA | 0 | 0 |
| 5 | E19-Dels | 0 | 3.1 | 2361 | 1755 | NA | ND | 0 | NA | ND | 0 | 0 | 0 | 0 |
|  |  | 143 | 3 | 4480 | 5644 | NA | 1340 | 89.3 | NA | 23.7 | 1.6 | 0 | 35.3 | 5.3 |
|  |  | 220 | 4 | 4970 | 2633 | NA | 946 | 76 | NA | 35.9 | 2.9 | 0 | 29.7 | 4.9 |
| 6 | E19-Dels | 0 | 3.3 | 1978 | 1771 | NA | 13.3 | 0 | NA | 0.8 | 0 | NA | 0.6 | 0 |
|  |  | 202 | 3 | 4600 | 4081 | NA | 38.7 | ND | NA | 0.9 | ND | 0 | 0 | 0 |
| 7 | E19-Dels | 0 | 3.8 | 4137 | 1795 | 0 | 93.7 | 37.9 | 0 | 5.2 | 2.1 | 0 | 4 | 0.5 |
|  |  | 39 | 3.2 | 5913 | 11929 | 0 | 192.5 | 36.3 | 0 | 1.6 | 0.3 | 0 | 1.3 | 0 |
| 8 | E19-Dels | 0 | 3.4 | 5529 | 8704 | 0 | 388.2 | 138.8 | 0 | 4.5 | 1.6 | 0 | 4 | 1.1 |
|  |  | 49 | 3 | 7987 | 6020 | NA | 1032 | 214.7 | NA | 17.8 | 3.6 | 0 | 15 | 5 |
| 9 | E19-Dels | 0 | 3 | 9920 | 14266 | NA | 1525.3 | 0 | NA | 10.7 | 0 | 0 | 14 | 0 |
|  |  | 172 | 3 | 6533 | 5950 | NA | 34.7 | 166.6 | NA | 0.6 | 2.8 | 0 | 4.6 | 1.1 |
| 10 | E19-Dels | 0 | 3 | 7987 | 3232 | NA | 58.7 | 0 | NA | 1.8 | 0 | 0 | 0.3 | 0 |
|  |  | 55 | 3 | 4760 | 2480 | NA | ND | 0 | NA | ND | 0 | 0 | 0 | 0 |
| 11 | E19-Dels | 0 | 3 | 26240 | 54677 | NA | 1088 | 3422.7 | NA | 2 | 6.3 | 0 | 7.5 | 4.8 |
| 12 | E19-Dels | 0 | 2.8 | 2700 | 2286 | NA | ND | 0 | NA | ND | 0 | 0 | 0 | 0 |
| 13 | E19-Dels | 0 | 3 | 60960 | 89214 | NA | 2141.3 | ND | NA | 2.4 | ND | 0 | 2.3 | 0 |
| 14 | E19-Dels | 0 | 2.7 | 2563 | 2673 | 0 | 0 | 0 | 0 | 0 | 0 | 0 | 0 | 0 |
| 15 | E19-Dels | 0 | 3 | 4333 | 3515 | NA | 0 | 0 | NA | 0 | 0 | 0 | 0 | 0 |
| 16 | E19-Dels | 0 | 3 | 4107 | 1941 | NA | 0 | ND | NA | 0 | ND | 0 | 0 | 0 |
| 17 | E19-Dels | 0 | 4 | 1630 | 1200 | NA | ND | 0.0 | NA | ND | 0 | 0 | 0 | 0 |
| 18 | E19-Dels | 0 | 3 | 2467 | 2757 | 0 | ND | ND | 0 | ND | ND | 0 | 0 | 0 |
| 19 | E19-Dels | 0 | 3 | 33427 | 225735 | 0 | 55821.0 | 8580 | 0 | 24.7 | 3.8 | 0 | 63 | 23 |
| 20 | E19-Dels | 0 | 3 | 10520 | 3907 | NA | 72 | ND | NA | 1.8 | ND | 0 | 2.3 | 0 |
| 21 | E19-Dels | 0 | 3 | 10493 | 4692 | NA | 78.7 | 0 | NA | 1.7 | 0 | 0 | 1.4 | 0 |
| 22 | E19-Dels | 0 | 3 | 7027 | 2399 | NA | 53.3 | 0 | NA | 2.2 | 0 | 0 | 1 | 0 |
| 23 | E19-Dels | 0 | 3 | 15453 | 7284 | NA | 2733.3 | 110.7 | NA | 37.5 | 1.5 | 0 | 33 | 1.4 |
| 24 | E19-Dels | 0 | 3 | 3680 | 2133 | NA | 0 | ND | NA | 0 | ND | 0 | 0 | 0 |
| 25 | E19-Dels | 0 | 3 | 9813 | 12385 | NA | 4346.7 | 438.7 | NA | 35.1 | 3.5 | 0 | 41 | 7.8 |
| 26 | E19-Dels | 0 | 3 | 5200 | 2726 | NA | 153.3 | 0 | NA | 5.6 | 0 | 0 | 1.6 | 0 |
| 27 | E19-Dels | 0 | 3 | 5733 | 3080 | NA | ND | 0 | NA | ND | 0 | 0 | 0 | 0 |
| 28 | E19-Dels | 0 | 3 | 11760 | 26265 | NA | 13426.7 | 0 | NA | 51.1 | 0 | 0 | 29 | 0 |
| 29 | E19-Dels | 0 | 3 | 4533 | 2249 | NA | 93.3 | 44 | NA | 4.1 | 2 | 0 | 4.7 | 0 |
| 30 | E19-Dels | 0 | 3 | 4760 | 2816 | NA | ND | 0 | NA | ND | 0 | 0 | 0 | 0 |
| 31 | E19-Dels | 0 | 3 | 2760 | 1640 | 0 | 0 | 0 | 0 | 0 | 0 | 0 | 0 | 0 |
| 32 | E19-Dels | 0 | 3 | 2333 | 1375 | NA | 29.3 | 0 | NA | 2.1 | 0 | 0 | 0 | 0 |
| 33 | E19-Dels | 0 | 1.8 | 4689 | 2249 | NA | 0 | 0 | NA | 0 | 0 | 0 | 0 | 0 |
| 34 | E19-Dels | 0 | 3 | 50253 | 114332 | NA | 10198.7 | 97.3 | NA | 8.9 | 0.09 | 0 | 14.1 | 0 |
| 35 | E19-Dels | 0 | 3 | 4827 | 2972 | NA | 0 | ND | NA | 0 | ND | 0 | 0 | 0 |
| 36 | E19-Dels | 0 | 3 | 4453 | 4219 | NA | 0 | 0 | NA | 0 | 0 | 0 | 0 | 0 |
| 37 | E19-Dels. T790M | 0 | 3 | 4960 | 14209 | 0 | 2706.7 | 209.3 | 0 | 19.0 | 1.5 | 0 | 29 | 1.3 |
| 38 | E19-Dels. T790M | 0 | 3 | 5200 | 4693 | NA | 1105.3 | 0 | NA | 23.6 | 0 | 0 | 23.8 | 0 |
| 39 | L858R | 0 | 3.3 | 4400 | 4195 | 0 | NA | 0 | 0 | NA | 0 | 0 | 0 | 0 |
|  |  | 202 | 3 | 4080 | 2611 | 2156 / 0 | NA | 735 | 39.2 / 0 | NA | 28.1 | 33 / 0 | 0 | 32 |
| 40 | L858R | 0 | 2.7 | 3407 | 2211 | 1554.1 / 0 | NA | 0 | 41.3 / 0 | NA | 0 | 39 / 0 | 0 | 0 |
| 41 | L858R | 0 | 3 | 7680 | 7053 | 0 | NA | 0 | 0 | NA | 0 | 0 | 0 | 0 |
| 42 | L858R | 0 | 3 | 20080 | 25911 | 7362.7 / 0 | NA | 928 | 21.5 / 0 | NA | 2.7 | 26 / 0 | 0 | 7 |
| 43 | L858R | 0 | 3 | 4400 | 2627 | 0 | NA | 0 | 0 | NA | 0 | 0 | 0 | 0 |
| 44 | L858R | 0 | 3 | 5560 | 4613 | ND | NA | 0 | ND | NA | 0 | 0 | 0 | 0 |
| 45 | L858R | 0 | 3 | 2627 | 1520 | 0 | NA | 0 | 0 | NA | 0 | 0 | 0 | 0 |
| 46 | L858R. T790M | 0 | 3 | 13613 | 28951 | 330.7 / 0 | NA | 73.3 | 1.4 / 0 | NA | 0.3 | 2 / 0 | 0 | 0 |
|  |  | 83 | 3 | 4227 | 2892 | 769.3 / 0 | NA | 269.3 | 26.6 / 0 | NA | 9.3 | 30 / 0 | 0 | 11 |
|  |  | 153 | 3 | 4187 | 2687 | 0 | NA | 0 | 0 | NA | 0 | 0 | 0 | 0 |
| 47 | L858R. T790M | 0 | 3 | 4693 | 3293 | 0 | NA | 0 | 0 | NA | 0 | 0 | 0 | 0 |
|  |  | 81 | 3 | 3560 | 1827 | 0 | NA | 0 | 0 | NA | 0 | 0 | 0 | 0 |
|  |  | 115 | 3 | 2480 | 1457 | 0 | NA | 0 | 0 | NA | 0 | 0 | 0 | 0 |
| 48 | L858R. T790M | 0 | 2.7 | 65956 | 125476 | 55022 / 0 | NA | 0 | 44/ 0 | NA | 0 | 53 / 0 | 0 | 0 |
|  |  | 127 | 2.9 | 29352 | 30610 | 871.7 / 0 | NA | 0 | 2.8 / 0 | NA | 0 | 0.7 / 0 | 0 | 0 |
| 49 | L861Q | 0 | 3.5 | 1737 | 3126 | 0 | NA | 0 | 0 | NA | 0 | 0 | 0 | 0 |
| 50 | L861Q | 0 | 4.9 | 2465 | 8437 | 0 / 2028.6 | NA | ND | 0 / 24.0 | NA | ND | 0 / 33 | 0 | 0 |
| 51 | EGFR WT | 0 | 3 | 57080 | 96707 | 0 | ND | 0 | 0 | ND | 0 | 0 | 0 | 0 |
| 52 | EGFR WT | 0 | 3 | 13227 | 28947 | 0 | ND | 0 | 0 | ND | 0 | 0 | 0 | 0 |
| 53 | EGFR WT | 0 | 3 | 8560 | 15947 | 0 | ND | 0 | 0 | ND | 0 | 0 | 0 | 0 |
| 54 | EGFR WT | 0 | 3 | 7853 | 4587 | 0 | ND | 0 | 0 | ND | 0 | 0 | 0 | 0 |
| 55 | EGFR WT | 0 | 3 | 1893 | 827 | ND | ND | 0 | ND | ND | 0 | 0 | 0 | 0 |
| 56 | EGFR WT | 0 | 3 | 3360 | 2283 | ND | ND | 0 | ND | ND | 0 | 0 | 0 | 0 |
| 57 | EGFR WT | 0 | 3 | 3227 | 2920 | 0 | 0 | 0 | 0 | 0 | 0 | 0 | 0 | 0 |
| 58 | EGFR WT | 0 | 3 | 3333 | 2827 | 0 | 0 | 0 | 0 | 0 | 0 | 0 | 0 | 0 |
| 59 | EGFR WT | 0 | 3 | 1640 | 2427 | ND | 0 | 0 | ND | 0 | 0 | 0 | 0 | 0 |
| 60 | EGFR WT | 0 | 3 | 4120 | 4067 | ND | ND | 0 | ND | ND | 0 | 0 | 0 | 0 |
| 61 | EGFR WT | 0 | 3.5 | 2891 | 5691 | 0 | 0 | 0 | 0 | 0 | 0 | 0 | 0 | 0 |
